# Supplementary material for: Zero shot molecular generation via similarity kernels
Source: Nat Commun. 2025 Jul 1;16:5991. doi: 10.1038/s41467-025-60963-3 (PMC12216838; doi:10.1038/s41467-025-60963-3)
Supplement: Supplementary file 1 — Supplementary Information [file 41467_2025_60963_MOESM1_ESM.pdf]

Supplementary Information: Zero Shot Molecular Generation via Similarity Kernels

S1. Similarity analysis of unconditionally generated molecules

To complement SMILES-based novelty and uniqueness assessments, we performed a Tanimoto similarity analysis using RDKFingerprints (roughly equivalent to Morgan Fingerprints [1] with radius 3, 2048 bits) as implemented in RDKit. For similarity-based uniqueness, we assessed the fraction of molecules forming unique clusters, where molecules within the same cluster have a Tanimoto similarity of ≥ 0.7 to its centroid. This 0.7 threshold is a common benchmark for chemical similarity searches [2]. For novelty, we defined molecules as novel if their maximum Tanimoto similarity to any molecule in the training set was below 0.7.

The inherently low similarity-based uniqueness observed for the QM9 dataset, as shown in Table I, is expected given its nature as an enumeration of small, closely related organic molecules.

S2. Validity metrics

Following Hoogeboom *et al.* [3], valid atoms were defined as those which had a number of nearest neighbours less than or equal to the natural valence of their element. Nearest neighbours correspond to atoms falling inside a particular atom's covalent radius.

Molecular validity was calculated either by checking if all atoms within a molecule are stable (for Figure 4) or by converting the generated point clouds into SMILES format using Open Babel [4] and checking if they can be sanitized using RDKit (Table I).

S3. Energy distribution of SiMGen generated molecules

To gain additional insight, we compared the similarity to reference environments (quantified by E_{sim}) and the energy evaluated using an accurate machine learning force field (ML-FF) of the generated structures. Figure S1 compares the cumulative similarity and energy distributions of molecules generated via SiMGen to 10,000 randomly sampled molecules from QM9 and 1,000 structures produced by the linear interpolation baseline (Section II A). Molecules generated with the similarity kernel exhibit almost identical similarity and energy distributions to QM9 molecules, suggesting our method successfully generates structures matching the reference distribution. In contrast, molecules from the linear scheme have higher energies and lower similarity to the reference data, likely due to the prevalence of fragmented and highly unsaturated structures.

S4. Analysis of guided generation with SiMGen

This section provides a detailed quantitative analysis of molecular generation with and without SiMGen guidance. To investigate the effects of different guidance strategies, we conducted several experiments in addition to the inverse sum guidance described in the main text. These included: first, employing the original direct sum similarity energy for guidance; and second, implementing a MolDiff inpainting approach (described in Section S5), designed to inherently incorporate the penicillin core structure. For each model configuration, we generated approximately 5,000 molecules, each targeted to contain 20 heavy atoms, with the exception of the MolDiff inpainting experiment, which we discuss separately below.

The results are summarized in two tables. Table S1 compares the impact of different guidance modes on the frequency of the targeted penicillin core fragments, as well as on the overall quality and diversity of the generated molecules. Table S2 further examines the influence of guidance on finer structural details, such as the distributions of bond lengths, angles, and torsions.

Looking at Table S1, we see that direct sum guidance had a negligible effect on the generated molecules. As previously discussed in the main text, the direct sum similarity energy (E_{sim}) is satisfied when each local environment in a generated molecule closely resembles at least one environment from the reference set. Consequently, a low energy state can be reached by matching some of the environments of the penicillin core, without generating the complete substructure. Furthermore, we found that employing direct sum guidance necessitated a substantially smaller guiding

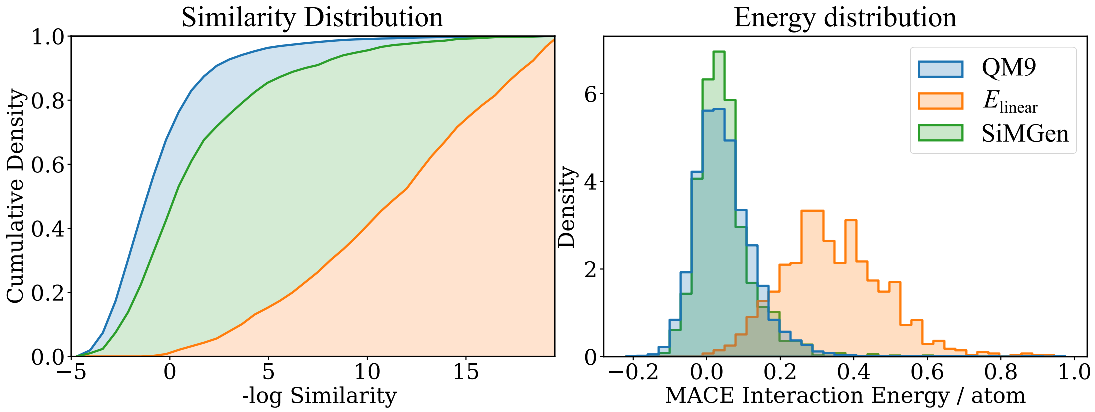

FIG. S1. **Molecules generated with the similarity kernel correctly sample the reference distribution** Molecules generated with the similarity kernel match the reference data both in energy and similarity. The E_{linear} baseline often generates fragmented and highly unsatured structures, resulting in high energy and low similarity. All structures were relaxed with a pretrained ML-FF before comparison. QM9 = samples taken from the QM9 [5] dataset, E_{linear} = samples generated with linear interpolation from a restorative potential to quantum mechanical energy. $n = 10000$ for the QM9 molecules, and $n = 1000$ otherwise.

coefficient compared to inverse sum guidance to prevent complete molecular fragmentation. This observation suggests that the gradient of the inverse sum energy function is more consistently aligned with the inherent score function learned by the generative model.

In contrast, inverse sum guidance demonstrably increased the propensity to generate molecules containing the targeted penicillin core substructures. Notably, this enhancement in targeted generation did not compromise the overall quality of the generated molecules, as evidenced by the consistent drug-likeness metrics compared to the unguided model. Interestingly, score guidance alone resulted in higher molecular diversity, indicated by a higher fraction of unique clusters, compared to the unconditional model. This effect can be attributed to the limited diversity within the GEOM-drug training dataset itself; generating molecules enriched with β-lactams effectively broadens the chemical diversity. The increase in diversity is visually supported by Figure 9B, which illustrates a wider distribution of generated molecules under score guidance. As expected, incorporating importance sampling reduced diversity by increasing the proportion of molecules with high similarity within the generated batch.

At first glance, MolDiff inpainting appeared to be the most effective method for generating structures incorporating the targeted fragments. However, these generated molecules exhibited the lowest drug-likeness scores and the highest Jensen-Shannon divergences from the unguided MolDiff baseline. It is also noteworthy that the fraction of structures successfully incorporating the targeted substructures was not 100%, despite the inpainting approach being designed to inherently include these fragments. Examination of the generation trajectories suggests that the trained MolDiff model often introduced additional atoms into either the four-membered or five-membered rings of the penicillin core. Indeed, during the inpainting process, the frequent generation of structures with overlapping atoms led to MolDiff's generation script triggering an early termination flag, as it repeatedly failed to construct valid SMILES graphs from the resulting geometries.

S5. MolDiff inpainting

To do inpainting with Moldiff, we follow the standard inpaiting procedure introduced by Lugmayr *et al.* [10]:

$$\boldsymbol{x}_{t-1}^{\text{known}} \sim \mathcal{N}\left(\sqrt{\bar{\alpha}_t}\boldsymbol{x}_0, (1-\bar{\alpha}_t)I\right) \tag{S1}$$

$$\boldsymbol{x}_{t-1}^{\text{unknown}} \sim \mathcal{N}\left(\mu_\theta(\boldsymbol{x}_t, t), \Sigma_\theta(\boldsymbol{x}_t, t)\right) \tag{S2}$$

$$\boldsymbol{x}_{t-1} = m \odot \boldsymbol{x}_{t-1}^{\text{known}} + (1-m) \odot \boldsymbol{x}_{t-1}^{\text{unknown}} \tag{S3}$$

Where $\bar{\alpha}_t$ is the training noising schedule, m is a mask, and $\mu_\theta(\boldsymbol{x}_t, t) - \frac{\boldsymbol{x}_t}{\sqrt{\alpha_t}} \sim s_\theta(\boldsymbol{x}_t, t)$. s_θ being the model's learnt score function.

For our penicillin case study, the known part corresponds to the β-lactam thiolane fused ring system.

Model	Observed Frequency of Targeted Substructures			Drug-likeness (↑)			Fraction unique clusters			Similarity to training data		
	Frac. β-lactam	Frac. Thiophene	Num. w/both	QED	SA	Lipinski	≥0.95	≥0.70	≥0.50	≥0.95	≥0.70	≥0.50
MolDiff	0.2% (1.0)	5.4% (1.0)	0	0.77	0.85	5.00	0.90	0.56	0.13	0.05	0.51	0.92
MolDiff (Inpainting)	13.7% (55.2)	32.0% (5.9)	36	0.68	0.57	5.00	1.00	0.73	0.10	0.00	0.20	0.90
Direct Sum Guidance IS ✗, Score ✓	0.1% (0.5)	9.6% (1.8)	1	0.78	0.87	4.99	0.92	0.55	0.14	0.04	0.58	0.93
IS ✓, Score ✗	0.2% (0.8)	6.4% (1.2)	0	0.77	0.85	5.00	0.77	0.56	0.17	0.04	0.45	0.89
IS ✓, Score ✓	0.2% (1.0)	8.7% (1.6)	1	0.75	0.85	4.98	0.80	0.56	0.17	0.03	0.45	0.89
Inverse Sum Guidance IS ✗, Score ✓	2.1% (8.3)	16.5% (3.1)	11	0.75	0.82	4.99	0.99	0.75	0.23	0.01	0.38	0.86
IS ✓, Score ✗	0.2% (0.8)	7.9% (1.5)	0	0.76	0.84	5.00	0.72	0.52	0.16	0.04	0.47	0.89
IS ✓, Score ✓	3.7% (14.9)	21.9% (4.0)	44	0.75	0.78	4.99	0.82	0.67	0.24	0.01	0.29	0.80

TABLE S1. Performance metrics for guided molecular generation, assessing substructure frequency, drug-likeness, and diversity/novelty. **Observed Frequency of Targeted Substructures:** Fraction of generated molecules containing a β-lactam or thiophene moiety, and the number of molecules containing both. Values in parentheses indicate the fold-change relative to the unguided MolDiff model. **Drug-likeness:** Quantitative Estimation of Drug-likeness (QED) [6], Synthetic Accessibility (SA) Score [7], and adherence to Lipinski's Rule of Five [8], calculated using RDKit [9]. **Fraction unique clusters:** Fraction of generated molecules forming unique clusters, where a cluster is formed by molecules with a Tanimoto similarity above the indicated thresholds to the cluster centroid. **Similarity to training data:** Fraction of generated molecules with a Tanimoto similarity above the indicated thresholds to the most similar molecule in MolDiff's GEOM-drug training dataset, i.e. inverse novelty.

Model	Jensen–Shannon distance			Fragmented Fraction
	Lengths	Angles	Torsions	
MolDiff	0.00	0.00	0.00	1%
MolDiff (Inpainting)	0.32	0.36	0.37	3%
Direct Sum Guidance IS ✗, Score ✓	0.07	0.05	0.04	2%
IS ✓, Score ✗	0.02	0.02	0.02	1%
IS ✓, Score ✓	0.10	0.08	0.08	3%
Inverse Sum Guidance IS ✗, Score ✓	0.19	0.15	0.18	8%
IS ✓, Score ✗	0.02	0.01	0.02	1%
IS ✓, Score ✓	0.22	0.19	0.22	8%

TABLE S2. Jensen-Shannon distances relative to unguided MolDiff and fragmented molecule fractions for different model configurations. Higher Jensen-Shannon distance indicates a greater difference in the distribution of bond lengths, angles and diherdral torsions.

S6. Shape of QM9 molecules

In Section II C, we showed that the choice of prior has a substantial effect on the final shape of generated molecule. As such, to generate QM9-like molecules we need to know what is the average molecular shape.

If $\sigma_1^2 \leq \sigma_2^2 \leq \sigma_3^2$ are the size-ordered variances along the principal axes of a molecule, then the covariance of a Gaussian prior that best fits the shape of the molecule is $\mathbf{\Sigma} = \mathrm{diag}(1, \sigma_2^2/\sigma_1^2, \sigma_3^2/\sigma_1^2)$. Note that the absolute values of the variances are not important because the volume of the prior is automatically scaled with the number of atoms.

Figure S2 shows the variance ratios for 10,000 randomly selected molecules from the QM9 dataset. Fitting a Gaussian kernel density estimate, we find that the distribution peaks at $(\sigma_2^2/\sigma_1^2, \sigma_3^2/\sigma_1^2) \approx (1.4, 2.6)$.

Thus, the molecular shape in the QM9 dataset is best approximated by a covariance $\mathbf{\Sigma} = \mathrm{diag}(1., 1.4, 2.6)$. Whenever QM9-like molecules were generated in the text, we used a prior with this specific covariance.

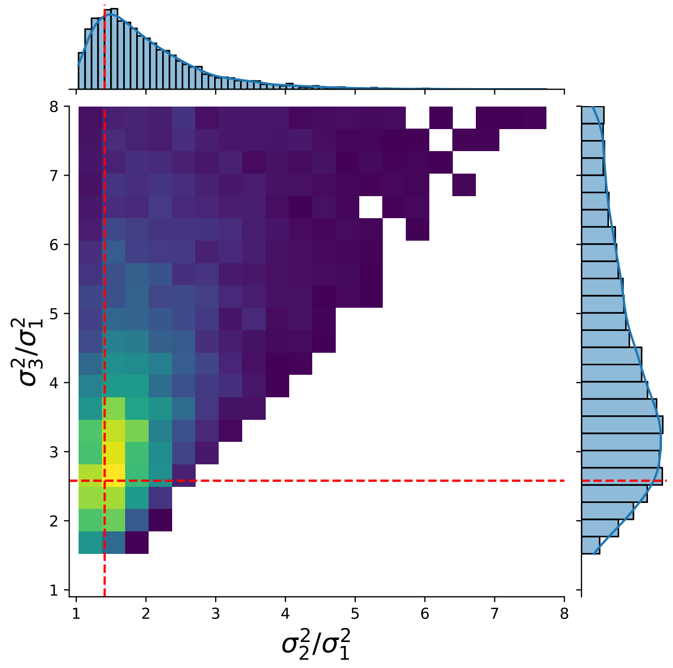

FIG. S2. **Shape of QM9 molecules** The axes correspond to variance ratios along molecular position principal axes. Based on kernel density analysis, the most likely variance ratio is (1.,1.4,2.6) which is also indicated by the red lines.

S7. ZnDraw graphical user interface

Figure S3 depicts ZnDraw's user interface and a linker generated with SiMGen using a user supplied point cloud prior. Most generation settings can be controlled via drop-down menus, including how many atoms to generate, the number of generation steps and the strength of the restorative prior.

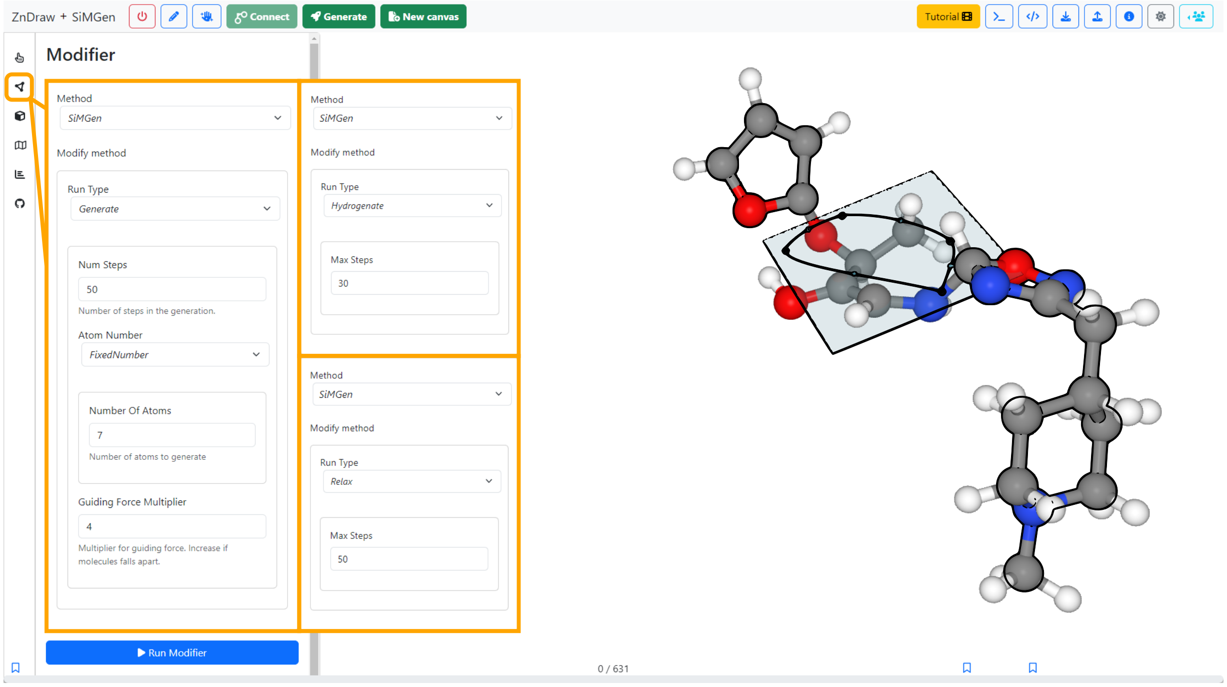

FIG. S3. **Graphical user interface of the ZnDraw package** The menus, highlighted in orange, allow user to adjust the number of generated atoms, strength of the attraction to the prior, and number of steps in the generation. The black outline on the molecule shows the original atom positions before generation. The points on the plane define the guiding point cloud for the generation.

Supplementary references

[1] D. Rogers and M. Hahn, Journal of Chemical Information and Modeling **50**, 742 (2010).
[2] Y.-C. Lo, J. Z. Torres, Y.-C. Lo, and J. Z. Torres, in *Special Topics in Drug Discovery* (IntechOpen, 2016).
[3] E. Hoogeboom, V. G. Satorras, C. Vignac, and M. Welling, "Equivariant Diffusion for Molecule Generation in 3D," (2022), arxiv:2203.17003 [cs, q-bio, stat].
[4] N. M. O'Boyle, M. Banck, C. A. James, C. Morley, T. Vandermeersch, and G. R. Hutchison, Journal of Cheminformatics **3**, 33 (2011).
[5] R. Ramakrishnan, P. O. Dral, M. Rupp, and O. A. von Lilienfeld, Scientific Data **1**, 140022 (2014).
[6] G. R. Bickerton, G. V. Paolini, J. Besnard, S. Muresan, and A. L. Hopkins, Nature Chemistry **4**, 90 (2012).
[7] P. Ertl and A. Schuffenhauer, Journal of Cheminformatics **1**, 8 (2009).
[8] C. A. Lipinski, F. Lombardo, B. W. Dominy, and P. J. Feeney, Advanced Drug Delivery Reviews Special Issue Dedicated to Dr. Eric Tomlinson, Advanced Drug Delivery Reviews, A Selection of the Most Highly Cited Articles, 1991-1998, **46**, 3 (2001).
[9] RDKit, online, "RDKit: Open-source cheminformatics," `http://www.rdkit.org`.
[10] A. Lugmayr, M. Danelljan, A. Romero, F. Yu, R. Timofte, and L. Van Gool, in *Proceedings of the IEEE/CVF Conference on Computer Vision and Pattern Recognition* (2022) pp. 11461–11471.
